# Prevalence of Dyslipidemia and Associated Factors in Obese Children and Adolescents

**DOI:** 10.4274/jcrpe.1867

**Published:** 2015-08-31

**Authors:** Selin Elmaoğulları, Derya Tepe, Seyit Ahmet Uçaktürk, Fatma Karaca Kara, Fatma Demirel

**Affiliations:** 1 Ankara Children’s Hematology and Oncology Training and Research Hospital, Clinic of Pediatric Endocrinology, Ankara, Turkey; 2 Ankara Children’s Hematology and Oncology Training and Research Hospital, Biochemistry Laboratory, Ankara, Turkey; 3 Yıldırım Beyazıt University Faculty of Medicine, Ankara Children’s Hematology and Oncology Training and Research Hospital, Clinic of Pediatric Endocrinology, Ankara, Turkey

**Keywords:** dyslipidemia, obesity, children, adolescents

## Abstract

**Objective:**

Childhood-onset obesity is associated with increased mortality and morbidity related to cardiovascular diseases (CVD) during adulthood. Dyslipidemia has a fundamental role in the pathogenesis of CVD. This study aimed to evaluate the prevalence of dyslipidemia and related factors among obese children and adolescents.

**Methods:**

Obese patients aged between 2 and 18 years were included in the study. Serum concentrations of total cholesterol (TC), triglyceride (TG), low-density lipoprotein (LDL-C), high-density lipoprotein (HDL-C), fasting glucose levels, insulin, thyroid-stimulating hormone (TSH), free thyroxine (fT4), alanine aminotransferase (ALT), aspartate aminotransferase (AST), and liver ultrasound findings were evaluated retrospectively.

**Results:**

Among 823 obese patients, 353 (42.9%) met the dyslipidemia criteria: 21.7% had hypertriglyceridemia, 19.7% had low levels of HDL-C, 18.6% had hypercholesterolemia, and 13.7% had high levels of LDL-C. Older age and/or high body mass index (BMI) were related to increased prevalence of dyslipidemia. Hepatosteatosis was more common among dyslipidemic patients. The frequency of insulin resistance (IR) and of higher levels of ALT and TSH were also detected in dyslipidemic patients. Patients with both dyslipidemia and grade 2-3 hepatosteatosis had higher levels of ALT, AST and TSH and lower levels of fT4.

**Conclusion:**

Prevalence of dyslipidemia is high in obese children, and hypertriglyceridemia is in the foreground. Higher levels of IR and more apparent abnormal liver function test results are observed in the context of dyslipidemia and hepatosteatosis coexistence. Metabolic and hormonal alterations related with thyroid functions may also be associated with dyslipidemia and hepatosteatosis in obese patients.

## INTRODUCTION

The worldwide prevalence of obesity in children has increased in the past 3 decades (1). It is estimated that there are 43 million overweight and obese children under 5 years of age worldwide (2). The prevalence of overweight and obesity in Turkish children is lower than that reported for North and South American countries and similar to North European countries (3).

Obesity is considered as an important public health problem by the World Health Organization (WHO) (4). It constitutes a major risk factor for cardiovascular disease (CVD), which is known to be the main cause of death and morbidity in adults. CVD-related symptoms generally appear in the fourth decade of life, but development of atherosclerosis is known to begin at earlier ages and to be related to dyslipidemia (5). In autopsy studies, it has been shown that fatty lines which are early signs of atherosclerosis can be determined even at the age of 2 and it has also been found that plaque thickness is proportional to age, body mass index (BMI), serum total cholesterol (TC), triglyceride (TG), low-density lipoprotein (LDL-C) and inversely proportional to high-density lipoprotein (HDL-C) (6). In addition to obesity and dyslipidemia, insulin resistance (IR) and high blood pressure, which are components of the metabolic syndrome, are also risk factors for CVD development (7). In order to decrease CVD-related death and morbidity in adulthood, obesity and dyslipidemia should be prevented in children and adolescents. In this retrospective study conducted on a large population of Turkish children, we aimed to evaluate the frequency of dyslipidemia and related factors in obese children and adolescents.

## METHODS

The records of cases aged between 2 and 18 years and diagnosed with obesity in the time period between 2011 and 2013 at Ankara Children’s Hematology-Oncology Training and Research Hospital Pediatric Endocrinology clinic were evaluated retrospectively. Data relating to age, anthropometric measurements, pubertal state, BMI, lipid profile, insulin resistance (IR), thyroid function tests, liver function tests, and presence of hepatosteatosis at admission were recorded from patient files, and the correlations between these parameters were assessed. Syndromic obese cases and cases with missing test results were not included in the study.

Body weight measurements were conducted using a “Barimed® Electronic Body Scale SC-105” with 0.1 kg accuracy, after a 10-hour fasting period, barefoot, and with daily clothes on. Heights were measured with “Ayrton® Stadiometer Model S100” with 0.1 cm accuracy, ≥barefoot. BMI was calculated using the kg/m2 formula. According to age and gender, a subject with a BMI value ≥95th percentile was considered as obese. The BMI percentile and BMI standard deviation (SD) values were calculated using the reference values developed by Neyzi et al (8).

Tanner staging was used for evaluation of puberty development. A testicle volume of ≥4 mL in males and presence of breast development Tanner stage ≥2 in females were accepted as signs indicating initiation of puberty (9,10).

Blood samples taken from patients after 8-10 hours of fasting were evaluated with standard methods using a Roche Modular-P 800 device. Fasting blood glucose, TC, TG, HDL-C, alanine aminotransferase (ALT) and aspartate aminotransferase (AST) levels were analyzed. LDL-C levels were calculated with available lipid data using the Friedewald formula (11). Serum TC levels over 200 mg/dL, TG levels over 150 mg/dL, LDL-C levels over 130 mg/dL, or HDL-C levels under 40 mg/dL were accepted as dyslipidemia (12,13). According to reference values of ALT and AST kits used in our hospital laboratories, ALT and AST normal values were set as below 41 U/L and 37 U/L, respectively.

Fasting insulin, thyroid-stimulating hormone (TSH), and free thyroxine (fT4) levels were studied with 2-chamber 2-step enzymatic immunoassay methods using a Beckman Coulter DxI 800 device. According to reference values of Beckmann Coulter TSH and fT4 kits used in our hospital laboratories, TSH low and high limit values were set as 0.34-5.6 mIU/mL and fT4 low and high limit values were set as 0.6-1.2 ng/dL.

IR was evaluated with homeostasis model of assessment for insulin resistance (HOMA-IR) index using the following equation: fasting insulin concentration (µU/mL) x fasting glucose concentration (mmol/L)/22.5 (14). For IR, HOMA-IR cut-off values were accepted as 2.5 in prepubertal and 4.0 in pubertal patients (15).

Upper abdominal ultrasonographic examination was used for diagnosis of hepatosteatosis in the radiology department of our hospital using a Toshiba Xarioi Style ultrasound device. Liver ultrasound findings were staged as follows: normal liver appearance (no hepatosteatosis), mild (stage 1), moderate (stage 2) and severe hepatosteatosis (stage 3) (16).

The subjects were classified into 3 groups according to evaluation of clinical and laboratory results of dyslipidemia and hepatosteatosis.

Group 1: Cases with both dyslipidemia and grade 2-3 hepatosteatosis

Group 2: Cases with dyslipidemia or hepatosteatosis

Group 3: Cases without dyslipidemia and hepatosteatosis

The study proposal was approved by the Ethics Committee of Ankara Children’s Hematology and Oncology Training and Research Hospital (Approval number: 2014-043).

### Statistical Analysis

Statistical analysis was performed using the Statistical Package for the Social Sciences version 17.0 (SPSS, Inc. Chicago IL, USA, Microsoft). Values were given as mean ± standard deviation (minimum-maximum). Student’s t-test was used to compare means of numeric variables, and chi-square test was used to compare non-numeric variables. One-way ANOVA test was used to compare numeric variables in groups in three (Post hoc: Bonferroni). Significance was accepted as p<0.05.

## RESULTS

The mean age of the 823 (459 female, 364 male) obese patients in the study was 10.8±3.1 years; 60.8% of them were pubertal. Demographic features of the patients and laboratory data are shown in Table 1.

In our study group, 353 (42.9%) patients met the dyslipidemia criteria: 21.7% of the patients had hypertriglyceridemia, 19.7% had low levels of HDL-C, 18.6% had hypercholesterolemia, and 13.7% had high levels of LDL-C. There was no statistical difference in the prevalence of dyslipidemia according to sex. Older age and/or high BMI were related with increased prevalence of dyslipidemia (p=0.047 and p=0.045, respectively) (Table 2). In pubertal obese patients, TG levels were higher and incidence of hypertriglyceridemia was higher than in the others (p<0.001 and p=0.006, respectively).

Among patients with dyslipidemia, 223 (63%) had hepatosteatosis, 102 (28.9%) had IR, 28 (7%) had high ALT levels, 23 (6.5%) had high AST levels, and 14 (3%) had hypothyroidism. Dyslipidemic patients had a higher proportion of hepatosteatosis, IR and higher levels of ALT and TSH when compared to the non-dyslipidemic group (p<0.05). fT4 and AST levels did not show any significant difference between these groups (Table 2). In patients with stage 1 hepatosteatosis, thyroid function tests were not affected, but ALT levels were higher as compared to patients without hepatosteatosis. On the other hand, ALT, AST, and TSH values were higher and fT4 was lower in patients with stage 2-3 hepatosteatosis (Table 3).

## DISCUSSION

In this study, dyslipidemia prevalence was found as 43% in 823 obese children and adolescents. In our study group, dyslipidemia was observed most frequently as hypertriglyceridemia. Frequency of dyslipidemia was related with older age and higher BMI. There are several studies reporting different dyslipidemia rates (17,18,19,20). Prevalence of dyslipidemia changed between 10.7% and 69.9% among obese children in different populations. In a previous report on Turkish children, Cizmecioglu et al (21) have reported a dyslipidemia prevalence of 42.9% in 112 school-aged obese children, a finding very similar to our results. Korsten-Reck et al (17) have reported a dyslipidemia incidence of 45.8% in 546 German children. Hypertriglyceridemia was found to be more common than high LDL-C and low HDL-C levels in both of the above studies. Frequency of dyslipidemia was reported as 69.9% in 2064 obese Iranian children; frequency of hypertriglyceridemia was reported in 49.9% of the children included in this study. A low HDL-C was reported in 60% of Mexican obese adolescents with metabolic syndrome (18,19). In 538 Chinese obese male children, hypertriglyceridemia prevalence was found to be 10.7% and hypercholesterolemia rate was 14.2% (20). Variations in reported prevalence rates can be due to dietary habits in different cultures, or to ethnicity, different inclusion criteria, BMI variation (also including overweight cases in some studies), and differences in dyslipidemia definition.

Dyslipidemia is more common in obese patients than in non-obese ones (22,23). In obesity, high amounts of free fatty acid (FFA) are released due to lipolysis. These FFAs lead to hypertriglyceridemia by inhibiting lipoprotein lipase in adipose and muscle tissues, in addition to increased production of very-low-density lipoprotein (VLDL) and TG in the liver. Degradation of TG-rich LDL-C and HDL-C caused by hypertriglyceridemia with hepatic lipase leads to increasing low LDL-C levels and decreasing HDL-C levels. Increased low LDL-C and decreased HDL-C levels are major factors for development of atherosclerosis and CVD (24,25).

The relationship between dyslipidemia and higher BMI values in our study is compatible with results reported in previous studies (26). Cut-off BMI-standard deviation score (SDS) for dyslipidemia was determined as 1.22 by Gong et al (20). However, since adipose and muscle tissue cannot be differentiated, BMI is thought to be an invaluable indicator for dyslipidemia or CVD (19,27). In one study evaluating CVD risk factors, BMI-SDS was shown as an effective indicator for hypertension and waist circumference SDS was shown to be an effective indicator for dyslipidemia (28).

A high amount of adipose tissue influences insulin release and functions through direct lipotoxic effect and leads to secretion of immune cytokines (29). Muscle cells which are an important reservoir for glucose become irresponsive to insulin in the presence of extreme amounts of FFA, TG and other lipid metabolites due to obesity and dyslipidemia (30,31). Compensatory hyperinsulinemia leads to increased VLDL, TG and FFA production and decreased HDL-C levels in the liver. Meanwhile, lipogenesis is increased in peripheral tissue due to IR (32). Due to this vicious cycle between dyslipidemia and IR, in our study, higher HOMA-IR values were detected in dyslipidemic patients, and the number of cases with IR was found to be significantly higher in this group. This relationship was also highlighted in some previous studies (33,34).

The most common cause of chronic liver disease in children is non-alcoholic fatty liver disease (NAFLD). In the past 20 years, the incidence of NAFLD has increased, in parallel with the increase in obesity. In obese individuals, because of dyslipidemia and IR, lipid intake and production in the liver overcomes lipid clearance, leading to development of hepatosteatosis. Steatosis is the first hit in the ‘two hit’ theory of NAFLD pathogenesis. Oxidant, inflammatory or toxic damage constitute the second hit and lead to hepatosteatosis and cirrhosis (35). Differentiation of steatosis, steatohepatitis, and cirrhosis can be distinctly done with pathological sampling. Intensity of hepatosteatosis can be evaluated with ultrasound. In our study, hepatosteatosis was detected in 56% of the patients and in 63% of dyslipidemic patients. Grade 2-3 hepatic steatosis rates were found as 22% in dyslipidemic cases and 14% in others. In cases with dyslipidemia, hepatosteatosis rate was significantly higher than in cases without dyslipidemia. According to results from various studies, NAFLD incidence is between 12% and 68% (20,36,37). These different results are thought to be related to subjectivity of diagnosis and staging of hepatosteatosis with USG and the wide range of BMI values and number of patients in these different studies.

Liver function tests are usually normal in children with hepatosteatosis. High ALT levels usually demonstrate development of steatohepatitis (38). ALT levels were found to be higher in the presence of hepatosteatosis or dyslipidemia compared to other patients in our group. In stage 2-3 hepatosteatosis, ALT levels were even higher, and in addition to ALT, AST levels were also increased.

In the studies evaluating changes in TSH level and thyroid function tests in obese children, TSH and free triiodothyronine levels were reported to be higher and fT4 levels lower compared to healthy controls (39,40,41). These changes occur usually within normal limits and are proposed to be a part of the adaptation process to obesity (41). Increase in leptin levels and cytokines secreted from adipose tissue causes increase in deiodinase activity and decrease in iodine uptake in thyroid tissue. Changes in thyroid function are reversible and thyroid function tests become normal after losing weight (42). Thyroid hormone is an important modulator in the lipid metabolism. It shows a hypolipidemic effect by regulating lipid synthesis and oxidation (43). Hypercholesterolemia and hypertriglyceridemia are known to develop in hypothyroidism (44,45). In our study, TSH levels were found to be higher in the presence of dyslipidemia as compared to the levels in obese cases without dyslipidemia. In a study by Santos-Palasios et al (46), thyroid functions and lipid profiles of 20783 adult patients were evaluated and even within the normal reference range, lipid profile was found to be disrupted with increasing TSH. In stage 2-3 hepatosteatosis, TSH levels were increased and fT4 were decreased in reference ranges in our study. This change was even more significant when there is dyslipidemia concurrently with hepatosteatosis. In two recent studies, increased TSH levels were found to be correlated with hepatosteatosis and high ALT levels (47,48). It is stated that this situation could be related to sick euthyroid syndrome (49). Dullaart et al’s study (48) has also shown that low thyroid functions within normal limits correlate to higher ALT levels in the presence of IR and metabolic syndrome.

In conclusion, studies have shown that dyslipidemia prevalence is high in obese children and that it increases with age. IR and hepatosteatosis are more frequent in dyslipidemic patients. If there is hepatosteatosis coexisting with dyslipidemia, changes in thyroid functions are more apparent. In the evaluation of obese children and adolescents with dyslipidemia, it should be remembered that clinical and metabolic problems related to obesity might be severe and should be monitored closely.

## Figures and Tables

**Table 1:**
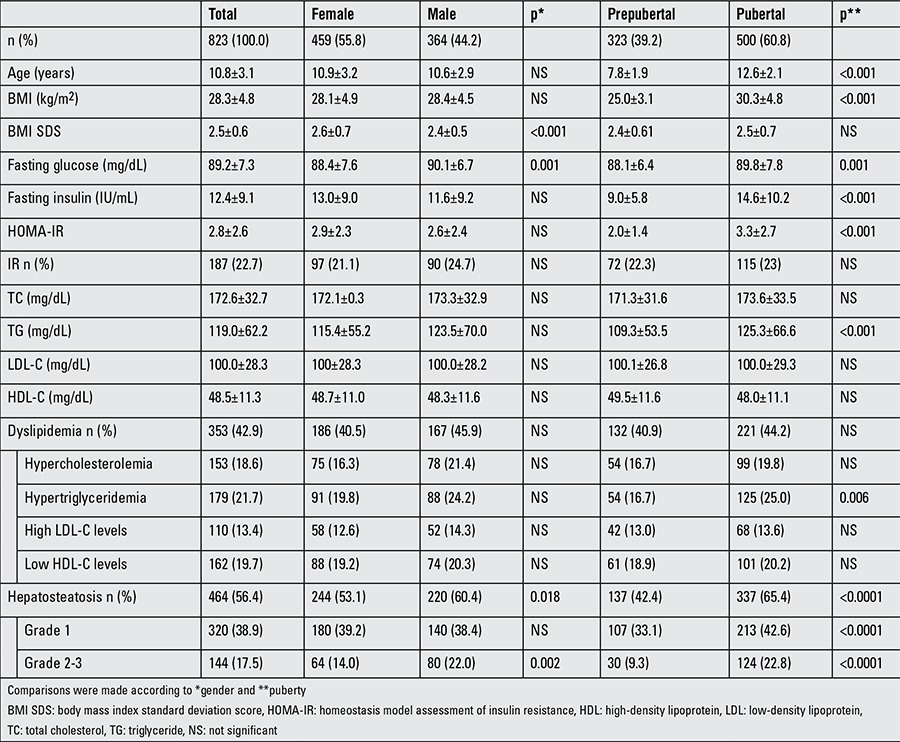
Demographic features of the patients and laboratory findings by gender and pubertal state

**Table 2:**
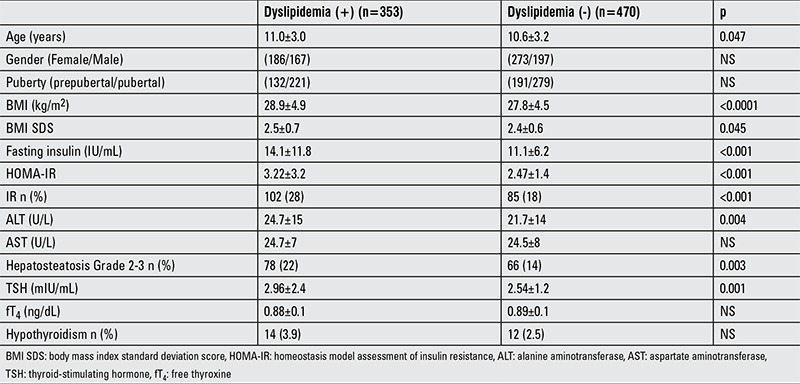
Clinical and laboratory differences between dyslipidemia (+) and (-) cases

**Table 3:**
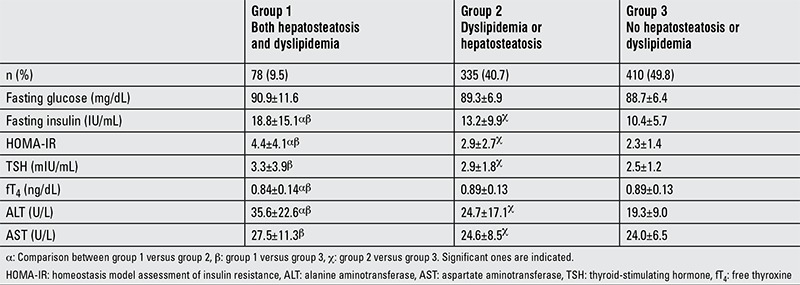
Comparison between laboratory findings in dyslipidemia and/or hepatosteatosis groups
